# Grid Plate Calibration at the National Bureau of Standards

**DOI:** 10.6028/jres.093.005

**Published:** 1988-02-01

**Authors:** Theodore D. Doiron

**Affiliations:** National Bureau of Standards Gaithersburg, MD 20899

**Keywords:** calibration, geometry correction, grid plate, measuring machine, metrology, two dimensional metrology

## Abstract

Grid plates are calibrated by a completely automated high precision measuring machine which uses a computer vision system to detect and locate the grid marks. The system routinely calibrates plates of up to 600 × 600 millimeters with accuracies of 0.5 micrometers. Descriptions of the system components, level of performance and tests of the absolute accuracy of the calibrations are presented.

## 1. Introduction

With the increasing use of video based dimensional measuring machines, there is a corresponding growth in the need for two-dimensional artifacts to use as calibration standards. Since the major uses for these systems range from measuring semiconductor geometry over a few square millimeters to photogrammetry and PC board inspections over a square meter, a system for calibrating such standards must have both high accuracy and a large dimensional range. The grid plates discussed in this paper are generally glass plates with some type of symmetric grid mark plated on the glass surface. The simplest type is a glass plate with a square grid of 20 *μ*m wide chrome lines spaced every one or two centimeters. However, the variety of materials and grid marks is large, ranging to metal plates with randomly drilled holes.

This note is designed to describe the theory, operation, and performance of the NBS automated grid plate calibration system. This system uses a computer controlled coordinate measuring machine and a computer based video system to detect and record the positions of the grid marks on grid plates up to 600 mm square. The normal calibration procedure is to measure the position of each grid mark on a plate in a pattern which repeatedly measures a few of the points several times distributed throughout the run to gather process control data on the calibration repeatability and drift. The run is then repeated. The plate is turned 90 degrees and measured twice again. This process of collecting redundant data provides enough information to give the positions on the grid marks, statistical information for realistic error estimates, and process control data about the measurement system.

The description of the system is organized into six sections: the coordinate measuring machine, the video imaging system, the measurement procedure, the data analysis, the system accuracy, and the outlook for upgrading the calibration performance.

## 2. Coordinate Measuring Machine

### 2.1 Hardware

The machine used for grid plate calibrations is a Moore Special Tool Company five-axis coordinate measuring machine^1^. It has a fixed bridge type geometry with a measuring volume of 1200×600×250 mm. The machine has been extensively studied and is described in detail in [1] and [2].

**Figure 1:**
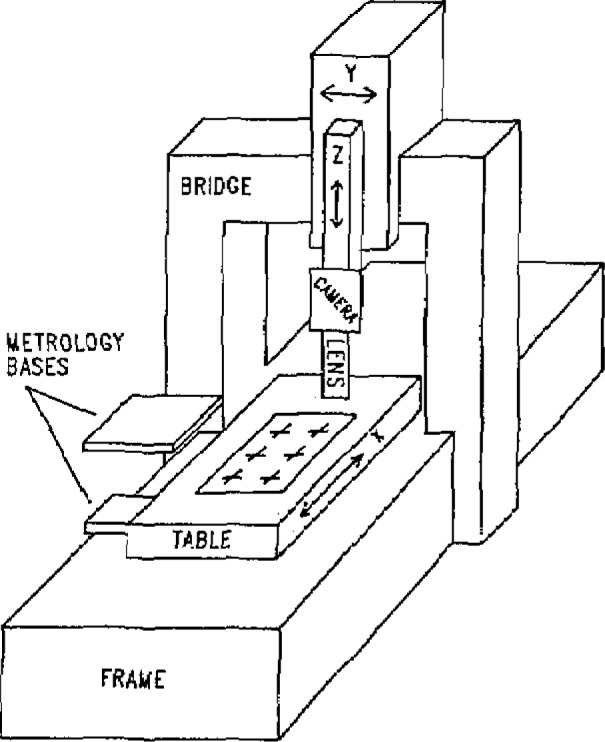
Diagram of coordinate measuring machine. The metrology bases support the laser interferometer systems. The error map relates the measured position of the bottom of the *Z* axis to the true position in an ideal reference frame.

The machine, shown in figure 1, has very accurate lead screws and guideways. To increase the accuracy even further, each axis was retrofitted with a laser interferometer which allows resolution of displacements of less than 0.02 *μ*m. The laser interferometer receivers and corner cubes were placed on metrology frames attached to the table, *Y* slide, and on top of the *Z* spindle. The *X* motion is provided by a table which travels on twin V roller bearing ways. The *Y* axis moves on twin V roller bearings on a fixed bridge. The total travel is 1200 mm in the *X* direction and 600 mm in the *Y* direction.

The geometric errors of the machine are quite small for a machine of this size. In order to obtain the maximum accuracy possible from the machine, a systematic study of the errors inherent in the machine was made.

While no attempt is made to correct for the small geometric errors in the machine motion during a measurement, software algorithms have been developed to correct the raw data for all of the known rigid body errors in the machine motion. A model of the machine was developed which included all of the rigid body systematic geometric errors, including the pitch, yaw, roll, and straightness of each axis. From very precise and repeated measurements of these errors an error map was developed which, when used with the model of the machine, could correct any measurement to an accuracy near the level of the repeatability of the machine.

**Figure 2:**
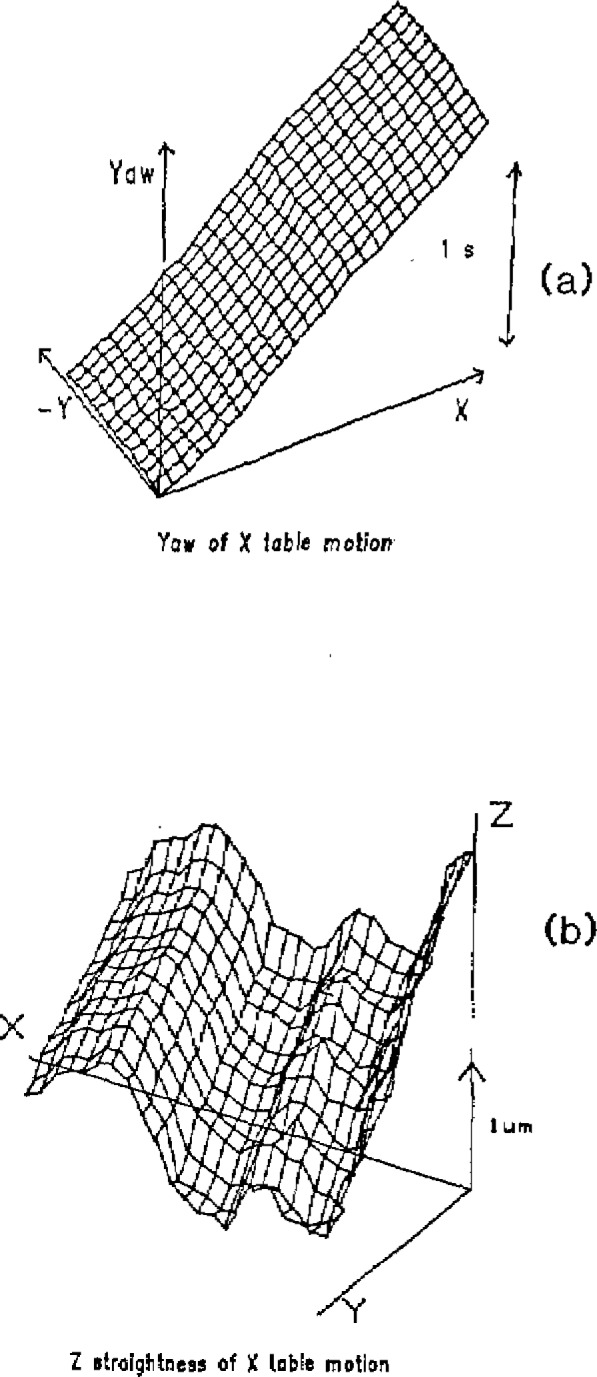
Typical error maps for coordinate measuring machine. The repeatability of the data is more than an order of magnitude smaller than the measured errors.

The error map was measured at each of 1950 equally spaced points in the measurement volume and the standard deviations of the measured errors were found to be quite small; about 0.01 second of arc for the angular error and 0.02 *μ*m for the straightness errors. Examples of the error diagrams obtained are shown in figure 2.

Many of the maps are smooth, as in figure 2(a). Even though the error map is measured at 2-inch intervals, a linear interpolation of the map provides an adequate measure of the error for points between measured points. Some of the errors are more complex, as in figure 2(b). It is obvious that there is structure to the errors between the calibration points which would result in small local systematic errors of up to 0.05 *μ*m. Since these interpolation errors in one cell of the calibration grid are uncorrelated with those of other cells, multiple measurements placing the grid at different places on the table will reduce the error by averaging.

The only significant systematic error which could not be adequately modeled is that due to the table bending. The machine moves on twin V cross section roller bearings. Because of small irregularities in the parallelism of the ways, the table is pinched slightly as it moves from one end of travel to the other. The table response is to bend upward in the middle, when unloaded, by as much as 2.5 *μ*m. Since this is not a rigid body motion it is difficult to include the associated errors in the model. However, because of the large number of correction parameters used in the model, some compensation is made in the fitting process and the residual error is small. The problem is serious only because the table sending depends on the weight and position of the piece being measured. For ball plates, which can be 50 to 200 kg, this is a serious problem as the error map might shift; for grid plates which weigh up to 1kg the problem is minimal.

Each time a calibration is made with the machine, the machine model and error map are used. By taking redundant data, as explained in section 4, any change in the error map will be apparent from the data analysis. Over the years since the original calibration was made, no significant change in the error map has been found.

### 2.2 Environmental Control

The machine environment, that is, temperature, air pressure and humidity, affect measurement accuracy. While the highest quality grid plates are made from quartz, which has a very small thermal expansion coefficient, the machine is made of steel, and the effects of environmental changes, on the measuring machine geometry are important. Besides maintaining the room temperature as nearly constant as possible, as many heat sources as possible have been controlled. For example:

- Each step motor has a temperature control system which circulates chilled water in tubing attached to the motor housing if the temperature rises above a preset temperature;
- The room has indirect lighting to prevent shadows;
- The measurement is completely automated so that no human heat source need be present near the machine after the run has begun;
- Ten thermocouples, placed at strategic locations on the machine, measure the thermal profile of the machine as the calibrations, are made to check on the actual thermal stability of the machine frame;
- Generally, the machine is run through a warm-up cycle for 2 to 6 hours to assure that thermal equilibrium is reached before the measurement run is begun.

The air temperature, pressure, and humidity are also important to the length scales of the machine. Since the interferometers measure in terms of the laser wavelength in air, the index of refraction of the air is important. Most laser measurement systems have available sensors which automatically make corrections, but in order to maintain the highest level of measurement assurance we measure the temperature, pressure and humidity independently and calculate the index of refraction from the formula of Jones [3].

**Figure 3:**
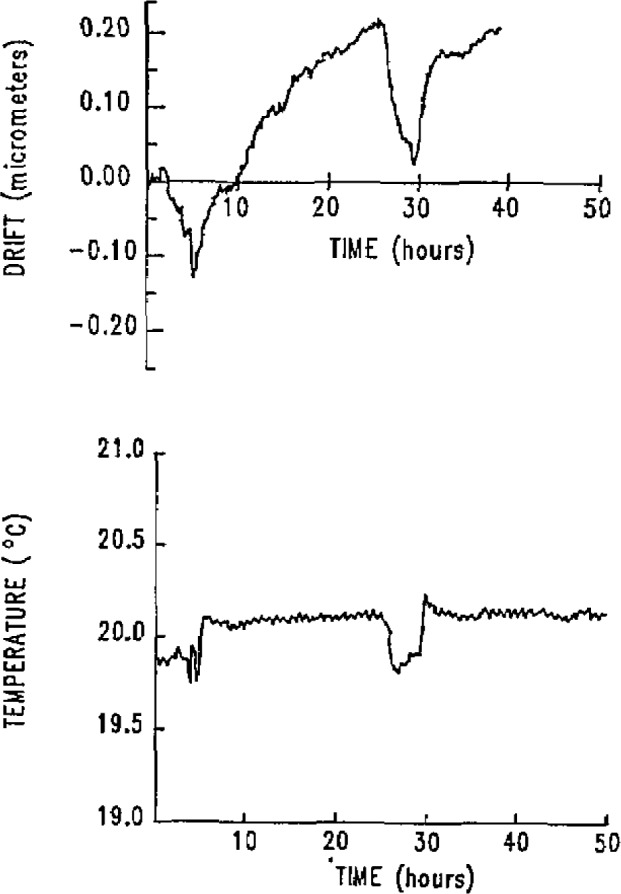
Room temperature and *Y* coordinate of the bottom of the *Z* axis as functions of time, showing machine response to change in room temperature.

The room stability is quite good over long periods of time, within 0.2 °C, but since the room is not separately thermostated, occasional temperature excursions occur. An example is shown in figure 3.

The top graph shows the *Y* direction motion of the bottom of the *Z* axis in response to the air temperature change shown in the bottom graph. The thermal time constant of the machine is over 2 hours, and four to six time constants are needed before the temperature stability is adequate for a calibration.

The temperatures of the air, grid plate, and machine are recorded at least every hour during the measurement to detect thermal problems. Because of the difficulty of developing a thermal model of the machine which would allow correction of the data for temperature changes, the machine thermal profile data is used only as a “go/no go” test for the calibration. If the machine thermal profile has been insufficiently stable during a particular measurement, that measurement is repeated. Even under the best conditions there is some small drift in the system over the 1 to 3 hours of a typical measurement. To compensate for some of this drift the measurement algorithm has at least one reference point which is repeated periodically during the run to provide a practical measure of the machine drift. Details of this technique are provided in section 4.

## 3. Video Imaging System

**Figure 4:**
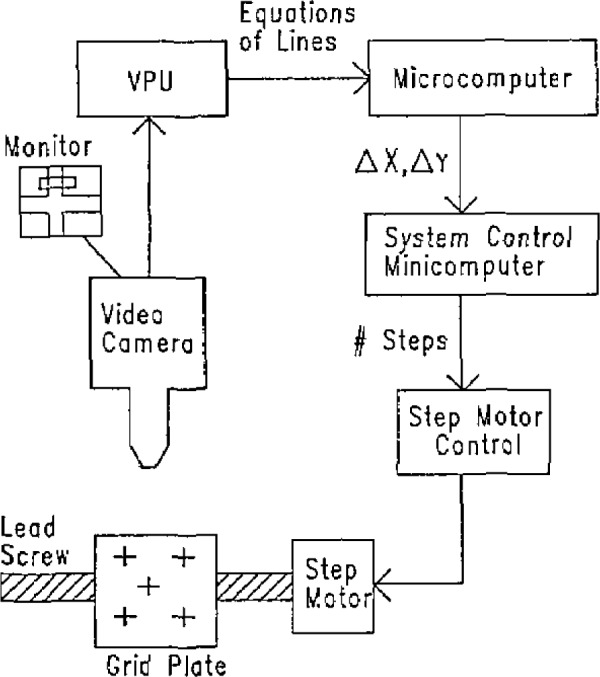
Block diagram of video system Feedback loop. The VPU finds the slope and intercept of each edge of the grid mark arms, using windows as shown on the monitor. The microcomputer functions as a smart interface and transfers position offset data to the system controller. The system controller moves the camera to the center of the grid mark.

A high-resolution television camera (vidicon) equipped with a commercial microscope system mounted on the *Z* axis of the machine is used to observe the marks (usually crosses) on the grid plate. A block diagram of the system is shown in figure 4.

The commercial video processor currently used is a simple edge detector. The output from a high resolution vidicon is fed into a processor and digitized into a 400×500 picture element (pixel) grid. The signal is compared with a user chosen intensity threshold, and on each line the positions of the first and last threshold crossings (dark to light or light to dark) are recorded in the system memory.

The raw data, or the results of any of a number of functions of the raw data performed in the processors firmware, can be output to a remote computer. Among the functions used for our measurements, the most important are:

Windowing—Any rectangular portion of the video image field can be used for analysis, ignoring the rest of the picture. By using windows, irrelevant lines and noisy areas of the picture can be avoided, allowing unambiguous analysis of the video data.
Centroid—The centroid of black or white pixels can be calculated. For symmetric grid marks such as crosses, Xs, and circles the centroid is used as a fast measure of the correction needed to move the mark close to the center of the screen.
Linear fit to an edge—A line can be fit to either the leading edge (first threshold crossing) data or the trailing edge data in any window. Thus, if the windows are chosen to include only one edge of a grid mark, the slope and intercept of the line can be obtained.

**Figure 5:**
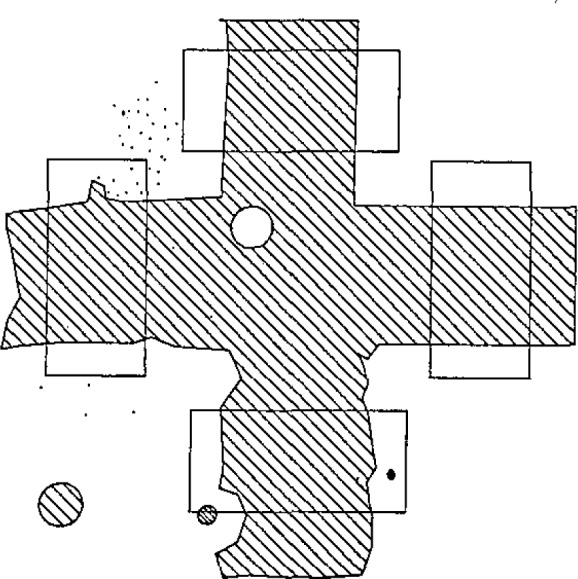
Composite grid mark showing placement of video windows and typical grid mark irregularities. The small dots represent video noise and dust.

There are several ways to define the center of the grid mark. Among the possibilities are the centroid of the grid mark, the centroid of the edges of the grid mark, and various fits to features of the mark. For most marks, which are crosses, we have chosen to fit a straight line to a section of the two edges of each of the four arms of the cross. Generally, an edge fit which avoids the actual intersections of the lines provides the most accurate calibrations, particularly for lower quality grids which have various irregularities at the line intersections. As shown in figure 5, there are a number of defects which can cause problems; such as dirt, which is removable, and chips and stray marks, which are not.

We use the centroid of the intersections of the eight lines as the center position. The difference between the calculated center of the grid mark and the center of the video display is used by the computer system to move the *X* and *Y* carriages until the camera is directly over the center of the mark as indicated by a null reading.

This procedure eliminates the need for precise calibration of the vision system. Since all of the grid marks are nearly identical, and the marks are brought to the same position and orientation in the video field, many systematic errors in the camera system are irrelevant. For example, since the image is symmetric, lowering or raising the threshold changes the width of the lines, but the apparent center is not displaced. Also, any small geometric distortion in the system optics does not affect the relative positions of the grid marks.

**Figure 6:**
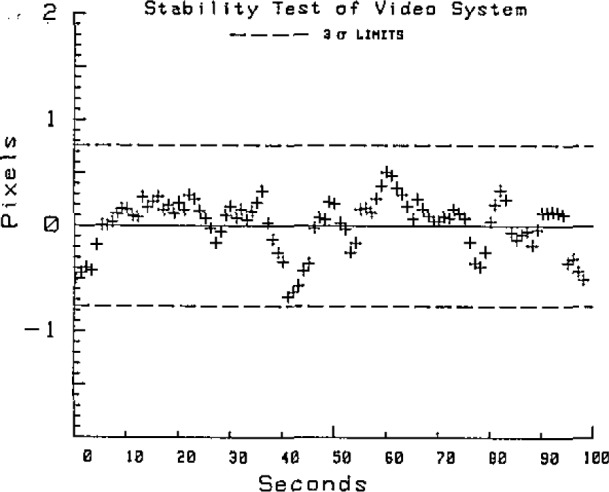
Stability and repeatability of the video system. The precision is relatively independent of the microscope magnification until diffraction from the edges becomes dominant.

Repeatability of the system is quite good. Multiple measurements of the center of a single grid mark gives a standard deviation of the calculated center coordinates of less than 1 pixel as shown in figure 6. For high quality grids with linewidths of 10 to 20 *μ*m, precision is commonly 0.2 to 0.4 pixels, and is nearly independent of the magnification of the microscope up to about 0.2*μ*m per pixel. At higher magnification diffraction effects become important.

One problem which has not yet resolved is that the algorithm for defining the center corresponds to that of a human operator only if the grid mark is smooth, sharp and free from defects. A human operator would ignore irregularities in the edge of the line. The current program uses these stray points as well as the edge points of the grid mark, a procedure which will produce calibrations with systematic differences from those of a human operator. Photoelectric microscopes have been used on many line centering systems [4,5] and are thought to provide a response close to that of a human observer. However, because of the large number of points used in the algorithm (over 1000) and the window placements, the lines must be quite defective to produce significant errors.

A number of other methods for defining the grid mark center have been tried, including algorithms using the centroid of the line rather than edge fits. Methods which use points on the interior of the lines, such as the centroid function, are less vulnerable to noise in the video signal but have lower precision than edge methods. Methods using multiple windows and various tests for edge irregularities can be used, but the amount of time needed for each intersection becomes much larger. Because of the increased time needed for the measurement, the gain in accuracy in the center measurement is usually canceled by random error due the variability of the room temperature. Plates which do not have consistent and well formed marks are assigned higher values of uncertainty.

For the highest quality grid plates, magnification is limited by diffraction; the edges of the lines become indistinct. This limit is reached for magnifications of more than 0.15 *μ*m per pixel. The effect of higher magnification is that the standard deviation of the measured coordinates becomes larger and cancels the gain in magnification. For most grid plates the optical magnification used is limited by practical considerations rather than limits due to diffraction or optical aberrations. The rules of thumb used to select the magnification depend on both the performance of the system and the characteristics of the grid plates calibrated in the past.

1. Geometric irregularities; e.g., widening or necking of the lines, chipped edges due to wear, or dirt are very frequent at the intersection of the two lines which make up the grid mark. Because of this a square area of two to three times the line diameter on a side centered on the grid mark center is avoided.
2. When the fit is done to the edges of the grid mark, there are two requirements for the fit. The first is that enough of the line is sampled to get a true picture of the line edge geometry. The second is that the fit must be accurate enough to be extrapolated to the center of the line to find the center.

Using these rules for 20 *μ*m lines (the most common size), magnification of approximately 0.5 *μ*m per pixel is used. For smaller lines more magnification, up to 0.15 *μ*m per pixel is used. The largest line encountered has been 250 *μ*m, and magnification of 2 *μ*m per pixel was necessary. At this low magnification the precision of the centering algorithm was seriously affected.

## 4. Measurement Procedure

**Figure 7:**
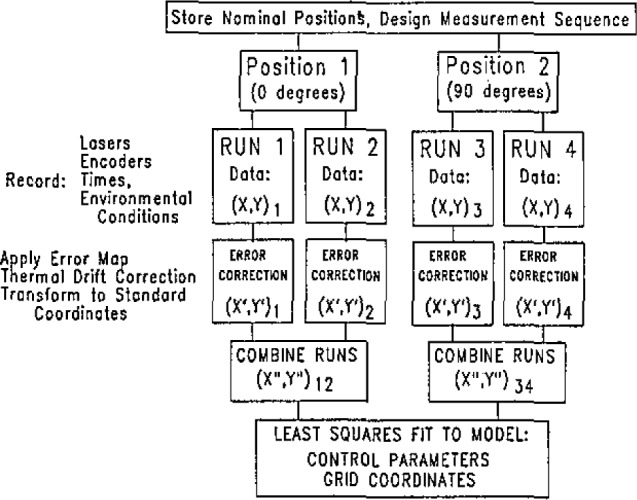
Overview of grid plate calibration system.

The measurement procedure and data analysis are designed to implement the principles of multiple redundancy and temporal modeling. There have been a number of schemes proposed [1,5–7]; the scheme currently implemented is that of Hocken and Borchardt [2]. An overview of the calibration is shown in figure 7.

The first step is to make up a measurement pattern, i.e., the order in which the points are to be measured. The program GRIDPLT controls the machine and records the coordinates, environmental factors, and the time at which each measurement is made. The raw data is then corrected for the known geometric errors of the machine by SHOWER2D. Thermal drift corrections, if needed, are also made in this program. Finally, the two runs in each orientation are combined and the data fit to the calibration model by ALBE3. This program reports the coordinates of the grid marks, the process control parameters, and an estimate of the calibration uncertainty. Each of these steps is explained in detail in the rest of this section.

**Figure 8:**
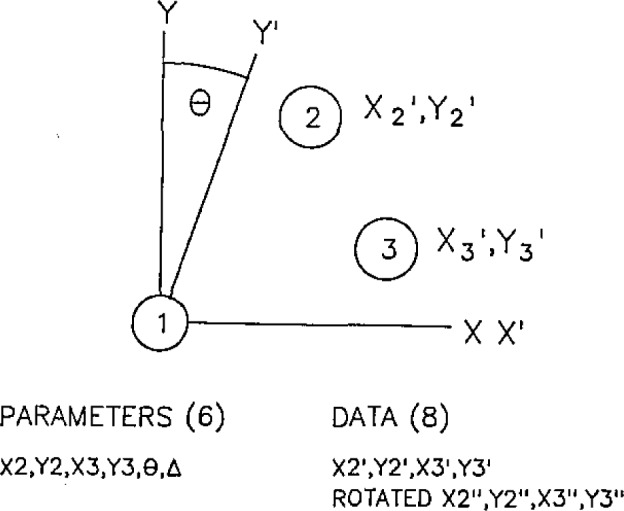
Simple model for three-point grid plate. In two runs, eight independent data points are taken. Since the model has six parameters, a best fit to the model can be made.

The model of the measurement system must include both the coordinates of the grid marks as well as all of the known systematic errors. For example, a grid plate model for the simplest case, three grid marks, might contain as parameters the true coordinates of the points, an angular correction for a possible error in the *X* and *Y* axis squareness, and a scale error for differences in the *X* and *Y* length scales. This case is shown in figure 8.

The true coordinates are in the ideal system *X* and *Y.* The machine coordinate system *X*′, *Y*′ is characterized by a possible difference in scale, Δ, and the deviation of the axes from orthogonality, Θ. Since the actual errors in the machine are small, a linear model is adequate.

The machine coordinates are set so that point 1 is the origin. If the coordinates of the points are measured in the machine coordinates, (*X*2′, *Y*2′) and (*X*3′, Y3′), and again with the plate rotated 90 degrees, (*X*2″, *Y*2″) and (*X*3″, Y3″), we have a total of eight data values. Since the model has only six parameters the model can be solved. Obviously for real grid plates with 12 to 130 points the number of redundant points is very large.

**Figure 9:**
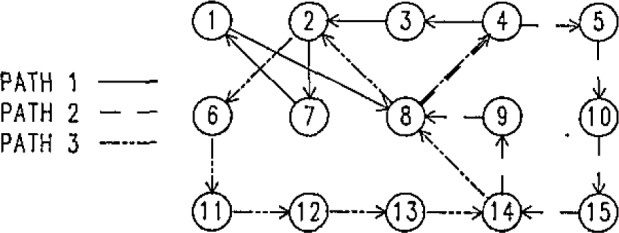
Typical calibration pattern. The complete design is path 1 followed by path 2 followed by path 3. Point 8 is the primary reference for measuring the drift in the machine geometry. The other repeat points are used to check the repeatability of the system.

In actual practice, the plate is measured four times, twice in one orientation and twice after rotating the plate 90 degrees. The two repeat runs are averaged to increase the precision of the coordinates. Each individual run has a number of repeated points which allow any drift in the machine coordinate system to be measured. A sample calibration pattern is shown in figure 9.

Notice that point 8 is taken several times during each run. The drift in the coordinates of point 8 is a measure of the drift in the machine coordinate system. To monitor the drift, the point must be taken at a frequency large with respect to the inverse time constant of the machine thermal relaxation. For most grids, one point measurement takes 2 to 3 minutes, and the repeat point is taken every five to 10 points to give a frequency of three to four times an hour. The thermal relaxation time is about 2 hours as mentioned in section 1.

**Figure 10:**
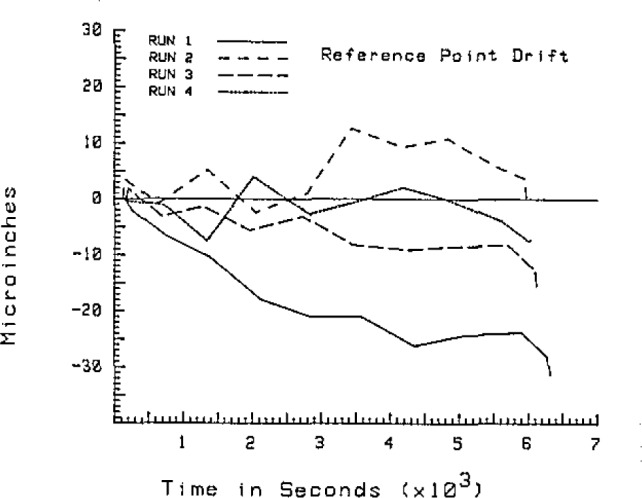
Time dependence of reference position during a typical calibration. Note that the longer the machine has been running the more stable the geometry becomes. Run 1 was preceded by a 2-hour warmup, and each run took about 1 hour.

At each point the coordinates and the time of the measurement are recorded. The currently used algorithm takes the change in the coordinates of the repeat point, assumes the change is linear in time, and applies a scaled correction to all of the points taken between the two measurements of the repeat point. A typical drift pattern is shown in figure 10.

Runs 2 and 4 are done directly after runs 1 and 3, respectively, and therefore have a longer effective warmup time. If the plate is turned directly after run 2, then runs 3 and 4 both have a long effective warmup. This usually leads to smaller drift corrections for runs 2 and 4, and sometimes 3 as shown in the figure 10.

Since the primary repeat point data is corrected for drift it has no apparent uncertainty in position. The standard deviations of the other repeat points from their average positions are pooled to get a quick measure of the repeatability of the run. If the repeat points are widely separated in time and space, this standard deviation is usually very close to the pooled standard deviation found when the two runs from one orientation are combined.

Using the technique of multiple redundancy in the data collection, (i.e., multiple readings of certain points in each run, plus multiple runs in multiple orientations), there is enough information to determine all of the model parameters. Since these parameters include both the coordinates of the grid marks and measures of the known systematic errors of the measurement system, each calibration provides measurement assurance data. With this constant monitoring of the major systematic errors, it is not necessary to make frequent measurements of our control grid plate to assess system performance.

The procedure also provides a measure of the unmodeled systematic errors since any errors which are not identical for both axes will increase the apparent random error of the calibration. In fact, the standard deviation of the combined four runs is generally about 0.03 *μ*m larger than that of the individual runs, implying small residual unmodeled systematic errors.

## 5. Performance

There are two important aspects of performance which have been studied; the precision and accuracy reported by the data analysis, and the true accuracy with respect to the national length standard.

About 50 plates calibrated with the system and the results have been very dependent on the quality of the grid. Table 1 shows the performance parameters for some of the plates run in the last 2 years. The uncertainty column gives the uncertainty derived from the final model fit of the combined data from the four runs. Since the analysis treats any unmodeled systematic error as a random error, and the uncertainty is taken as three times the apparent random error, this error estimate over estimates the true error.

The last plates M2607A and M2569 are plates with 250 *μ*m wide lines. After the four runs of the standard calibration scheme the uncertainty was over 2 *μ*m, a result of the loss of precision inherrent in the low magnification. Since the repeatability of the video system is near 1 pixel (3 *σ*) over a large range of magnifications, lower magnification leads to lower precision in setting the camera over the grid. For normal magnifications, the uncertainty is limited primarily by the accuracy of the coordinate measuring machine, 0.3 to 0.6 *μ*m depending on the thermal behavior of the room during the measurements. Only for very low magnifications is the video system the main source of uncertainty. For these plates the calibration was run again and the data combined. This reduced the random component of the error to an acceptable size.

The overall experience with the system shows that the uncertainty in the calibration is not strongly dependent on the size of the plate, number of points or the size of the grid line. When plates have over 100 points the length of the run becomes long (over 4 hours), and thermal drift becomes a problem. For lines over 40 *μ*m wide there is loss of precision in the video centering, but more runs can reduce the error. Plates over 600 mm on a side cannot be calibrated with the normal analysis software because the machine has only 600 mm of travel in the *Y* direction. For plates over 300 mm square there is an increase in run time due to the speed of the machine, 200 mm/min, which can allow thermal problems to affect the calibration repeatability.

The best quality grid plates which have been calibrated, JSB01 and JSB24, were calibrated as a test of the absolute accuracy of the system. The plates were first calibrated on the NBS linescale interferometer, a one-dimensional machine which is capable of calibrating any linescale less than one meter in length. The system is described in detail elsewhere [6,8]. Any line of grid marks on a grid plate of the type used here, less than 15 cm on a side, can be calibrated with uncertainties of less than 0.02 *μ*m. Since this accuracy is nearly an order of magnitude better than the Moore 5 grid plate system, the lines on a grid calibrated on the linescale can be treated as primary length standards. The grids were then recalibrated using the grid plate system.

One change in the normal grid plate software was made for these tests. Since the linescale is a one-dimensional instrument, only two arms of the grid marks are measured. For example, in the grid mark of figure 5, the linescale interferometer would use only the top line of the grid marks for the calibration in one direction, and the right line when the plate was turned to do the orthogonal direction. This measurement will be the same only if all of the grid marks have the same orientation to the measurement axis. In order to prevent any systematic errors due to irregularities in the alignment of the crosses, the video system was programmed to measure only the same two arms of the grid marks that were used by the linescale. Since the grid plate software demands that both arms of the grid mark be on the screen at the same time only a 40 *μ*m length of the line could be used in the fit. Since the linescale used up to 100 *μ*m there might be very small systematic errors due to edge deformities in the edge section not measured by the video system. This effect was not investigated.

To compare the calibrations, the distance between every two points on each line was calculated. For example, for line one of plate one there are nine grid marks one half inch apart. From this line there are eight half inch subintervals, 7 one inch subintervals, 6 one and one half inch subintervals, 5 two inch subintervals, etc. The differences between the grid plate system and the linescale calibrations were averaged by subinterval length and the pooled standard deviation from the average was found for each length. The results are given in tables 2 and 3.

The grid plate analysis program reported a pooled uncertainty of 0.33 *μ*m for plate JSB01. The deviation of the coordinates from the linescale calibration yielded a three times pooled standard deviation of 0.035 *μ*m, a good agreement with the analysis program result.

The same comparison for plate JSB24 found the analysis program uncertainty to be 0.45 *μ*m and the comparison with the linescale calibration gave a 3 *σ* of 0.38 *μ*m. Both of these results show that the grid plate analysis results are valid estimates of the absolute uncertainty of the calibration.

**Figure 11:**
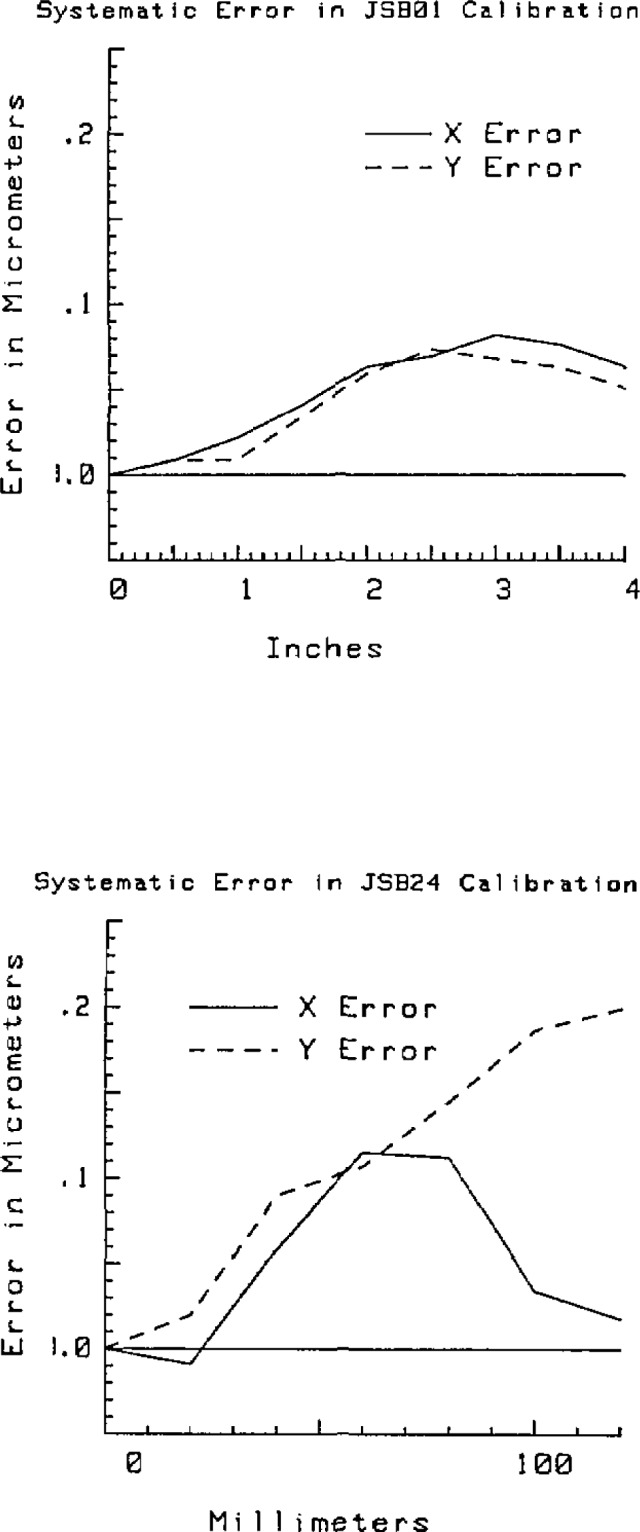
Systematic error in the calibration of two grid plates. These plots show the average difference between the two-dimensional grid plate system and the linescale interferometer measurements.

The length dependence of the errors are shown in figure 11. The systematic length dependent part of the error is different for the two plates. The most reasonable explanation of this effect is a result of the small scale errors in the machine geometry. Since the error map interpolates between the data at 2 inch intervals, nonlinear errors in the machine with characteristic length of less than 2 inches will not be corrected. This hypothesis is supported by fact that the characters of the error curves are different. Since the two plates were calibrated at different positions on the machine table, if the small scale errors not contained in the error map are the determining factor the error curves should vary with position on the table. Had the error curves been very similar an error source independent of the small scale geometric errors of the machine, for example the laser wavelength corrections or temperature scale, would be suspect.

It is possible to reduce this source of error by measuring the plate in random positions on the table, thereby averaging out the geometric errors. From tables 2 and 3 it can be seen that the random errors as estimated by the three times the pooled standard deviation are somewhat larger than the systematic errors as determined by the comparison to the linescale interferometer data, some gain might be made.

## 6. Outlook

Given the current performance level of the machine and the known limitations of the calibration method, there are a number of changes which would improve the accuracy and uncertainty of the grid plate calibrations.

The video system, under best conditions, has a reproducibility of about 0.02 *μ*m (1 *σ*). This level has been found for a number of small grid plate runs, where the thermal properties of the machine were very stable and the calibration run limited to 10 to 15 minutes. Increasing the speed of the machine motion, the video processing, and the thermal stability of the system could make this precision routine for most grid plates of less than 6 inches square.

If the error map were perfect, combining runs with different orientations or positions on the table would not increase the measured random error. The unmodeled systematic error in the machine, measured by the difference between the pooled standard deviations of the runs in the same orientation and the pooled standard deviation found when all of the runs combined, is generally 0.03 to 0.05 *μ*.m. This error is probably irreducible without physical changes in the machine.

Currently under study are plans to upgrade the performance of the machine by a major refit of the control system and improvement of the way geometry to reduce the nonrigid body motions of the machine. With added speed and small improvements in the thermal stability of the room, it may be possible to produce calibrations routinely at a level closer to the combination of the minimum errors of the separate system components, about 0.15 *μ*m.

## Figures and Tables

**Table 1:** Performance parameters.

Calibration	# pts	Size of plate (mm)	Total uncertainty (*μ*m)
E0079	67	200×200	0.64
M2470	133	230×230	0.80
M2463	121	200×200	0.67
M0001	13	200×200	0.36
M0002	13	100×100	0.74
M2340	12	170×100	0.48
M2341	12	170×100	0.70
JSB01	81	115×115	0.33
JSB24	45	120×120	0.45
M2607A	25	600×600	1.12
M2569	25	600×600	0.84

**Table 2:** Plate JSB01

Interval length (inches)	Average difference (mm)		Pooled standard deviation (*μ*m)
	*X*	*Y*	
0.5	0.01	0.01	0.09
1.0	0.02	0.01	0.09
1.5	0.04	0.04	0.10
2.0	0.06	0.06	0.08
2.5	0.07	0.07	0.08
3.0	0.08	0.07	0.07
3.5	0.08	0.06	0.06
4.0	0.06	0.05	0.05

**Table 3:** Plate JSB24

Interval length (mm)	Average difference (mm)		Pooled standard deviation (*μ*m)
	*X*	*Y*	
20	−0.01	0.02	0.22
40	0.06	0.09	0.31
60	0.12	0.11	0.24
80	0.04	0.13	0.25
100	0.03	0.19	0.24
120	0.02	0.20	0.26

## Footnotes

1 Certain commercial equipment, instruments, or materials are identified in this paper in order to adequately specify the experimental procedure. Such identification does not imply the recommendation or endorsement by the National Bureau of Standards, nor does it imply that the materials or equipment are necessarily the best available for the purpose.

## References

[1] Hocken RJ, Borchardt BR (1979). On Characterizing Measuring Machine Geometry. Natl Bur Stand (US) NBSIR.

[2] Hocken RJ, Simpson J, Borchardt B, Lazar J, Reeve C, Stein P (1977). Three Dimensional Metrology. Ann CIRP.

[3] Estler W Tyler (1985). High-accuracy displacement interferometry in air. Appl Optics.

[4] Beers JS, Lee Kang B (1982). Interferometric measurement of length scales at the National Bureau of Standards. Precis Eng.

[5] Loewen EG, Burns RH (1971). An Interferometric Scale and Grid Comparator. Ann CIRP.

[6] Reeve C (1979). A Method of Calibrating a Two Dimensional Reference Plates.

[7] Raugh MR (1985). Absolute two-dimensional sub-micron metrology for electron beam lithography. Precis Eng.

[8] Doiron T (1984). The Calibration of Gridplates at the National Bureau of Standards.

